# Complete Genome Sequence of *Streptococcus pneumoniae* Virulent Phage MS1

**DOI:** 10.1128/genomeA.00333-17

**Published:** 2017-07-13

**Authors:** Witold Kot, Mourad Sabri, Hélène Gingras, Marc Ouellette, Denise M. Tremblay, Sylvain Moineau

**Affiliations:** a Department of Environmental Science, Aarhus University, Roskilde, Denmark; b Département de Biochimie, de Microbiologie et de Bio-informatique, Faculté des Sciences et de Génie, Québec, Canada; c Groupe de Recherche en Écologie Buccale, Faculté de Médecine Dentaire, Université Laval, Québec, Canada; d Centre de Recherche en Infectiologie du Centre de Recherche du CHU de Québec and Département de Microbiologie, Infectiologie et Immunologie, Faculté de Médecine, Université Laval, Québec, Canada; e Félix d’Hérelle Reference Center for Bacterial Viruses, Faculté de Médecine Dentaire, Université Laval, Québec, Canada

## Abstract

The lytic *Streptococcus pneumoniae* phage MS1 was isolated from a throat swab of a patient with symptoms of upper respiratory tract infection. The genome of this siphophage has 56,075 bp, 42.3% G+C content, and 77 open reading frames, including queuosine biosynthesis genes. Phage MS1 is related to pneumococcal phage Dp-1.

## GENOME ANNOUNCEMENT

*Streptococcus pneumoniae* is a major human pathogen causing a diverse array of respiratory and invasive infections. Because antibiotic resistance has increased among strains of *S. pneumoniae*, lytic phages and endolysins are currently being reconsidered as alternatives to antibiotics. Despite the isolation of several pneumophages in the past (1), only three virulent pneumococcus phages are readily available through phage collections, Dp-1 (*Siphoviridae* family) as well as Cp-1 and SOCP (*Podoviridae* family) (2–5).

Here, a new virulent pneumococcal phage was isolated from a swab sample collected at the Centre Hospitalier de l’Université Laval (Quebec City). Briefly, the swab sample was eluted, filtered (0.45 µm), and propagated on *S. pneumoniae* strain R6 using brain heart infusion (BHI) broth supplemented with 5 ng/mL choline chloride (BHI-fc) and incubated overnight at 37°C (2). Several phage plaques were obtained and based on the DNA restriction profile of their genome, only one distinct phage was isolated, purified, and designated MS1. Genomic DNA of phage MS1 was isolated using a Lambda maxi kit (Qiagen) from a lysate. Genome sequencing was performed on a 454 FLX instrument (IBIS, Université Laval). The genomic sequence was completed by primer walking and the sequencing of several PCR products. The 17,814 raw reads (total of 6,821,949 bases) were assembled into one contig using the GS De Novo Assembler (Roche), with an average coverage of 122-fold. Genomic termini were determined using PhageTerm (6). Phage MS1 has a circularly permuted genome and uses headful (*pac*-type) packaging system. The genome of the virulent pneumococcal phage MS1 has a low G+C content (42.3%), and is composed of 56,075 bp and 77 genes. No tRNA genes were found.

Phages were washed with ammonium acetate (0.1 M, pH 7.5), stained with uranyl acetate (2%), and observed under an electron microscope. Observations revealed an icosahedral capsid of 67 ± 2 nm in diameter with a noncontractile tail of 179 ± 12 nm in length, indicating that phage MS1 belongs to the *Siphoviridae* family.

Gene prediction and annotation were performed using customized RASTtk (7) workflow with GeneMark (8). Automated annotations were verified with Glimmer’s predictions (9) and manually curated. MS1 genome is syntenic to *Streptococcus* Dp-1 phage (2), with an average nucleotide identity (ANIb) 73.3% on 62.3% of aligned nucleotides calculated using JSpeciesWS (10). Putative functions of gene products were assigned based on results found by BLASTP, HHpred, pfam (v29.0), and TMHMM (v2.0) searches (11–14). Promoters and terminators were predicted using BPROM and ARNold, respectively (15, 16). No known toxins or remnants of the lysogeny module were identified in the MS1 genome. Interestingly, phage MS1 also possesses queuosine biosynthesis genes, which have been found in a few other phage genomes (2, 17–19).

Phage MS1 was deposited at the Félix d’Hérelle Reference Center for Bacterial Viruses (http://www.phage.ulaval.ca) under the number HER 532.

### Accession number(s).

The complete genome sequence of phage MS1 is available in GenBank under the accession number KY629621.
